# An audit of families with unreported or misreported cancers verified through a population-based cancer registry: implications for providing cancer risk assessment and management advice by a Familial Cancer Centre

**DOI:** 10.1186/1897-4287-10-S2-A42

**Published:** 2012-04-12

**Authors:** M Kentwell, M Bogwitz, L Donoghue, T McArdle

**Affiliations:** 1 Royal Melbourne Hospital, Family Cancer Centre, Melbourne Health, Australia; 2 University of Melbourne, Masters Genetic Counselling student program, Australia; 3 Genetic Health Services Victoria, Australia; 4 Victorian Family Cancer Registry, Cancer Epidemiology Centre, Cancer Council Victoria, Australia

## Background

The Cancer Council Victoria manages a population-based cancer registry, the Victorian Cancer Registry (VCR). All cancer diagnoses in the state of Victoria are notified to this register. As part of the genetic cancer risk assessment process, the Victorian Familial Cancer Centres (FCCs) submit the family history pedigree to the Cancer Council for verification. Each person in the pedigree is checked against the Register. Cancer verification and pathology reports are returned to the FCCs within five days of the submitted request.

Since 2002 over eight thousand families have been submitted to the Cancer Council for verification with over 160,000 individuals checked for cancer. 3% of all individuals with no report of cancer had a cancer confirmed, and 4% had a different diagnosis confirmed to what was reported.

## Aim

To review the effect on genetic cancer risk assessment, including eligibility for genetic testing, and clinic management advice following verification of cancers by the Cancer Council in families seen at Melbourne Health FCCs during 2010 where:

1) An unreported cancer in a family member is confirmed.

2) A reported cancer in a family member is confirmed for a different cancer site.

## Method

Three hundred and fifty-six family pedigrees were submitted to the Cancer Council from Melbourne Health FCC for cancer verification during 2010. Of these, 135 families had a one or more cancers confirmed where no cancer was reported and, 89 families had a different cancer confirmed to the one reported by the proband.

Each of these pedigrees was reviewed, and data was collected on cancer risk assessment before and after the cancer verification information was obtained. For breast and ovarian cancer families, genetic testing eligibility scores were assessed using BRCAPRO. For bowel and uterine families, Immunohistochemistry (IHC) testing eligibility was assessed using the Revised Bethesda criteria.

## Outcomes

This presentation will summarise data including cancer risk assessment, BRCAPRO scores, Immunohistochemistry eligibility before and after the cancer verification for a subgroup of families verified in 2010. This data will inform how the Cancer Council verification process informed the subsequent clinical advice provided to patients and families as a result of confirmed cancer information. Several individual case studies of interest will be presented.

